# Two-stage analyses of sequence variants in association with quantitative traits

**DOI:** 10.1186/1753-6561-5-S9-S53

**Published:** 2011-11-29

**Authors:** Jennifer H Barrett, Jérémie Nsengimana

**Affiliations:** 1 Section of Epidemiology and Biostatistics, Leeds Institute of Molecular Medicine, University of Leeds, Cancer Genetics Building, St. James’s University Hospital, Beckett Street, Leeds, LS9 7TF, UK

## Abstract

We propose a two-stage design for the analysis of sequence variants in which a proportion of genes that show some evidence of association are identified initially and then followed up in an independent data set. We compare two different approaches. In both approaches the same summary measure (total number of minor alleles) is used for each gene in the initial analysis. In the first (simple) approach the same summary measure is used in the analysis of the independent data set. In the second (alternative) approach a more specific hypothesis is formed for the second stage; the summary measure used is the count of minor alleles in only those variants that in the initial data showed the same direction of association as was seen overall. We applied the methods to the simulated quantitative traits of Genetic Analysis Workshop 17, blind to the simulation model, and then evaluated their performance once the underlying model was known. Performance was similar for most genes, but the simple strategy considerably out-performed the alternative strategy for one gene, where most of the effect was due to very rare variants; this suggests that the alternative approach would not be advisable when the effect is seen in very rare variants. Further simulations are needed to investigate the potential superior power of the alternative method when some variants within a gene have opposing effects. Overall, the power to detect associations was low; this was also true when using a more powerful joint analysis that combined the two stages of the study.

## Background

Genome-wide association studies focus on genetic loci with common minor allele frequencies (MAFs > 0.05) and are not designed to detect the effects of rare variants. However, rare variants contribute to complex diseases and might be detectable using resequencing data. Several methods for analyzing this type of data have been proposed [1-4], but this research area is still in development and many questions remain unanswered. Given the cost burden of sequencing a large number of subjects across the entire exome or the whole genome, we postulate that in a typical study a relatively small sample can be analyzed comprehensively, following up on regions of interest in a second data set. The test statistic threshold in the first stage should not be too restrictive, to avoid false-negative results, while the second stage threshold should meet stringent criteria, to minimize false-positive results.

Using the simulated Genetic Analysis Workshop 17 (GAW17) data [5], we conducted analyses of quantitative traits Q1, Q2, and Q4 to evaluate the efficacy of this approach. We compared two approaches, one testing for overall evidence of association with the gene exactly as in the first analysis and the other forming a more specific hypothesis based on the initial results. Once the original simulation model was disclosed, we determined power and false-positive rates.

## Methods

### Simple strategy

For each sequence variant *i* in gene *j*, let *x_ijk_* be the number of copies of the minor allele (defined as the allele with population allele frequency less than 0.5) carried by individual *k* (so *x_ijk_* = 0, 1, or 2). Suppose that gene *j* includes *n_j_* sequence variants; then a summary measure for gene *j* in individual *k* is formed by counting the number of minor alleles:(1)

For each replicate, we randomly split the sample into an initial set of 349 (50%) subjects and an independent set consisting of the remaining 348 subjects. We tested association between each of the three simulated quantitative traits Q1, Q2, and Q4 and each gene in the initial set using a linear regression model, regressing the trait on *X_jk_* and adjusting for population in three categories (156 Europeans [CEPH population or Tuscan], 321 Asians [Chinese or Japanese], and 220 Africans [Luhya or Yoruba]). Genes showing evidence of association with the trait at a significance level of 0.01 were carried forward to the second stage of the analysis.

Suppose that, for a particular trait and replicate, *N* genes were carried forward to the second stage. These genes were then tested in the same way in relation to the trait, using the remaining data for that replicate. Genes were regarded as showing association in this second stage if they met the Bonferroni-corrected significance level of 0.05/*N*. Each analysis (first and second stage) was repeated for each trait in the 200 replicates.

### Alternative strategy: more specific hypothesis

We investigated an alternative strategy in which the *N* genes identified in the initial data set were tested in a different way in the remaining samples. Instead of summing all minor variants in the gene, we used information on which variants were positively or negatively related to the trait in the initial data set. Specifically, for each of the *N* genes, the direction of association between the sum of the variants and the trait in the initial set was ascertained (i.e., whether they were positively or negatively correlated). If they were positively correlated, we identified the subset of individual variants that were positively related to the trait in this data set (i.e., positive regression coefficient but not necessarily statistically significant).

Let *I_ij_* be an indicator variable such that *I_ij_* = 1 if variant *i* is positively related to the trait and *I_ij_* = 0 otherwise (including for variants not observed in the data set). We calculated a new summary measure for each gene, summing over just these variants:(2)

In the second stage, we tested association with the trait by regressing the trait on , adjusting for population as before. Similarly, we analyzed negatively related variants if the gene and the trait were overall negatively correlated.

As in the simple analysis, we regarded genes as showing association in this second stage if they met a significance level of 0.05/*N*. Results are presented for each quantitative trait for each gene that showed consistent evidence of association in the first-stage analysis (in at least 120 of the 200 replicates). Results are also presented for the genes that were simulated to be associated with each trait. Analyses were conducted using programs written for this purpose in R (R Development Core Team, Vienna).

### Comparison with joint analysis of the two-stage design

We also considered the increase in power that could be obtained by carrying forward the samples from the first stage into the final analysis, that is, by carrying out a joint analysis of the total data set for the *N* genes passing stage 1 but applying a Bonferroni correction for the original number of 3,205 genes. For this design (with an equal number of samples in the two data sets and an initial significance level of 0.01), Skol et al. [6] has shown that this correction approximately preserves the overall type I error rate.

## Results

### Q1, Q2, and Q4

Table 1 lists the 12 genes that show association with Q1 at a significance level of 0.01 in the first stage of the analysis in at least 120 replicates. The *FLT1* gene reaches this level of significance in 195 replicates, indicating a power of 97.5% with a sample size of only 349. The next two columns in Table 1 give the number of times a significant association is seen in the follow-up analysis using the simple approach and using the alternative strategy based on a more specific hypothesis. For most genes the performance of the two strategies is similar, but association is detected considerably fewer times for *KDR* using the alternative strategy compared to the simple strategy (39 vs. 75 replicates; see later discussion).

**Table 1:** Analysis of genes in relation to trait Q1 using the simple strategy, the alternative strategy based on more specific hypotheses, and the combined analysis with appropriate adjustment for multiple testing

				Number of significant replicates		
				
Gene	Chromosome	Number of variants in gene	Number of times passing stage 1 threshold	Simple strategy	Alternative strategy	Joint analysis
***FLT1***	**13**	**35**	**195**	**188**	**187**	**195**
*OR2T34*	1	16	166	79	77	140
***KDR***	**4**	**16**	**165**	**75**	**39**	**146**
*PRR4*	12	34	159	57	60	117
*JAK1*	1	12	150	49	45	105
*CYP4F3*	19	12	149	37	38	97
*TAS2R48*	12	12	148	41	53	102
*ZNF91*	19	9	145	45	42	87
*HLA-B*	6	28	143	33	36	89
*INSR*	19	16	138	42	39	91
*LOC645118*	19	5	125	24	21	73
*CES1*	16	30	123	28	32	64

Only genes significant in the first analysis (at the 1% level) in at least 120 of the 200 replicates are shown. The two genes in bold were simulated to be associated with the trait. All analyses are adjusted for population stratification.

In the list of our 12 top findings for Q1 (Table 1), 10 are false positives. With the exception of *PRR4*, all these genes are on the list of 695 genes found by others to be consistently spuriously associated with the simulated disease affection status, which was correlated with Q1 [7]. These genes were highly significantly more correlated with the single-nucleotide polymorphisms (SNPs) simulated to be causal than were the other genes in the data set [7].

The final column in Table 1 shows for comparison the number of times the joint analysis of the whole data set from the replicate is significant after adjusting for 3,205 tests and given that the gene passes the stage 1 analysis threshold. It can be seen that this joint comparison method has considerably more power than either of the other approaches, in agreement with what has been shown previously [6].

Seven other genes associated with Q1 in the simulation model were not detected in our analysis using any approach. Although all met the stage 1 significance level of 0.01 in at least two replicates (ranging from 2 for *HIF3A* to 28 for *VEGFC*), they were never significant in the follow-up analysis after applying the Bonferroni correction in the simple or alternative method. (With the joint analysis, only *FLT4* ever reached significance and this happened in only one of the 200 simulations.)

All 13 genes simulated to be associated with Q2 passed the initial threshold in at least one replicate (ranging from 1 in 200 replicates for *PLAT* and *PDGFD* to 70 in 200 replicates for *VNN1*). However, using the simple two-stage method, only *VNN1* ever reached our criterion for significance in stage 2 (in 10 replicates, among the 70 in which it passed stage 1), and only *VNN1* and *SIRT1* ever reached significance under the alternative method (12 out of 70 times and 1 out of 22 times, respectively). Even when carrying out the joint staged analysis, *VNN1* was significant in only 20 simulations, and no other gene was significant in more than 3 simulations. For this trait, power to detect associations was very low, and there were few false positives.

For trait Q4, no genetic effect was simulated. Because of the Bonferroni corrections applied, we would expect to find one gene meeting the final significance threshold in any particular replicate 5% of the time. In both the simple two-stage and joint analyses, the total number of significant findings in 200 replicates was 9 (experiment-wise error rate of 4.5%), which is consistent with expectation. This suggests that the strategies have correct statistical type I error rates and that the false-positive results observed for Q1 are a result of correlation between genes.

### Determinants of power to detect association

**Figure 1:**
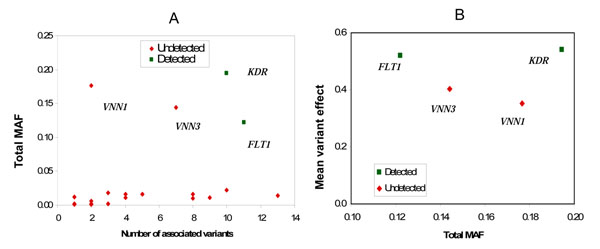
**Properties of the associated variants within a gene**. (A) Sum of MAFs of associated variants within each of the simulated genes (9 genes for Q1 and 13 genes for Q2) as a function of the number of associated variants. (B) Mean simulated variant effect within the four genes with highest total MAF.

Using any strategy, we detected only a small number of the genes that were simulated. As shown in Figure 1A, the two genes we identified with highest power (*FLT1* and *KDR*) contain 11 and 10 associated variants, respectively, with a total MAF greater than 0.1. Only two other associated genes, *BCHE* and *SREBF1*, have as many associated variants (13 and 10, respectively), but for these genes the total MAF of the associated variants is still low. The next most frequently detected gene was *VNN1*, which, along with *VNN3*, is one of only two other genes (both associated with Q2) for which the total MAF of the associated variants exceeds 0.1. Figure 1 shows not only that these genes contain fewer variants than the two genes detected but also that the average variant effect (simulated regression *β* coefficient) was smaller in these genes.

**Figure 2:**
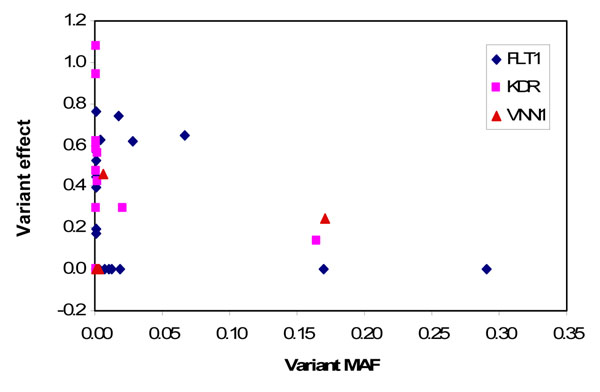
Simulated variants effect (regression *β* coefficient) in relation to their MAFs within *FLT1*, *KDR*, and *VNN1*

Unsurprisingly, the critical gene characteristics determining the power to detect an effect are the number of associated variants per gene, their total MAF, and the size of individual variant effects. However, the power to detect *KDR* was remarkably lower than the power to detect *FLT1* (Table 1), despite a similar number of variants (10 vs. 11), a higher total MAF of associated variants (0.19 vs. 0.12), and a comparable mean variant effect (0.54 vs. 0.52; see Figure 1). Figure 2 plots each variant effect within *FLT1*, *KDR*, and *VNN1* (simulated regression *β* coefficient) against its frequency. The *KDR* variants with strong effects (*β* > 0.3) are all extremely rare, in fact with no more than three instances of each allele observed in the total data, whereas three of the *FLT1* variants with strong effects are much more common. The rarity of the *KDR* variants has a drastic effect on power, although this is less marked in the combined analysis.

The two strategies for follow-up analysis performed similarly for *FLT1* and *VNN1* but not for *KDR*. This is likely again to be because most of the associated variants in *KDR* are rare; the variants that are observed in the initial data set and that are the basis of the “specific hypothesis” of the alternative strategy may not be observed at all in the second data set. Conversely other rare variants seen only in the second data set will not be included in the test because of lack of initial information on their relevance.

## Discussion and conclusions

The ability to resequence the genome or exome opens up new possibilities for the discovery of trait-associated variants whose frequencies are too rare for them to be likely to be discovered using genome-wide association studies. However, sequencing a large number of samples is likely to remain expensive for the foreseeable future. We considered ways in which such approaches might be made more efficient by applying two-stage strategies. In our approach, only a small fraction of the genes (on average 140 [4.4%] for Q1, 50 [1.6%] for Q2, and 34 [1.1%] for Q4) would have to be resequenced in the second set of samples. We investigated whether further efficiency savings or possible improvements in power could be made by considering only certain variants within those genes, on the basis of the direction of association in the initial sample. We expected that the method based on specific variants might improve power by removing from the gene’s summary statistic the noise variants that were not related to the trait (or even variants having an opposing effect); we also expected that power would be lost if variants were present in the training data set that were not found in the test set (private or very rare variants). Except in the case of one gene (*KDR*), the alternative method worked just as well as the simple strategy, even though less genotyping was required because fewer variants needed to be analyzed. Our analyses were conducted blind to the simulation models, but in fact in these models minor alleles were only ever associated with *increases* in trait values. Clearly, in this situation the alternative method we considered would lose some of its potential advantages. Variants with no relationship to the trait would still be removed from the summary statistic (with probability 0.5), but there would be no variants with opposing effects to remove.

Because in the GAW17 data the genotypes were unchanged across replicates, we selected both the training and the retest data sets from within one replicate. Of course, if extensive sequence data were available on a sample of size 697, analysis of the whole cohort making use of all data in a single stage would be considerably more powerful than any two-stage strategy, but our purpose was to assume that the cost of large-scale sequencing was the main limitation to increasing sample size. The joint analysis showed that, even with a staged design, with consequent savings in sequencing effort, if data were available on the selected genes from both stages, then it would be more powerful to incorporate all the data into the final analysis. However, there would still be a limitation in that few of the truly associated variants would pass the initial threshold. Power to detect rare variants was low with a sample size of 349, unless the total MAF of all the associated variants was high and the effect size strong enough. It would therefore be desirable to use a considerably larger sample size than this for the hypothesis-generating stage of a study, although it can be reasonably assumed that the cost of sequencing will stay high for some time, which makes the availability of extensive sequence data in large samples unlikely. In the meantime, efforts must be devoted to the development of novel, more powerful methods. A recent study [8] highlights the interest in two-stage designs, including a more in-depth sequencing of some genes or regions in the final stage.

The method we used to summarize the variants within a gene in the simple strategy included all minor alleles in the gene, which is unlikely to be the most powerful approach. In the alternative strategy (summing over specific variants), the approach we used to restrict the analysis to variants showing a beneficial (or deleterious) effect while reducing the impact of neutral variants was similar in spirit to the data-adaptive summation method of Han and Pan [9] (see also Dering et al. [10]). Various other approaches to reduce the number of variants tested have been proposed, such as setting a threshold allele frequency, including only nonsynonymous SNPs, or applying weighting schemes. Our purpose was to compare the specific-variant strategy with an analysis that does not utilize the information from the first-stage analysis; this strategy could easily be adapted to include further constraints on the variants, or weighting schemes, to derive alternative summary measures.

The simple strategy out-performed the alternative strategy for one gene, for which only very rare variants had a strong effect, suggesting that the alternative approach would not be advisable where the effect is mainly attributable to very rare variants. Further simulations are needed to investigate the potential superior power of the alternative method when some variants within a gene have opposing effects. Consistent with previous findings, joint analysis of the stage 1 and stage 2 samples with appropriate correction for multiple testing is more powerful than either of the methods in which the follow-up data set is analyzed separately; this suggests that if raw data for the initial samples are available, joint analysis is the preferred method. It would be of interest to apply the alternative specific-variant method to joint analysis of the two stages, but it would be nontrivial to adjust the significance level appropriately (without complex simulation), because the stage 1 samples would be used both to define and to test the hypothesis.

## Competing interests

The authors declare that there are no competing interests.

## Authors' contributions

JHB carried out the main programming and analyses; JN carried out some analyses and produced tables and figures. Both authors designed the study and wrote the manuscript.
